# Between Vulnerability and Deservingness: Social Imaginaries About Migrants Among Primary Health Care Nurses

**DOI:** 10.3390/healthcare14172803

**Published:** 2026-09-01

**Authors:** Ángel Martín-García, Cristina Oter-Quintana, Milagros Rico-Blázquez, Fernando Jesús Plaza del Pino, María Idoia Ugarte-Gurrutxaga

**Affiliations:** 1 San Blas Health Centre, Primary Care Management, Madrid Health Service, 28981 Parla, Spain; 2 Department of Nursing, Faculty of Medicine, Autonomous University of Madrid, 28049 Madrid, Spain; cristina.oter@uam.es; 3 Instituto de Investigación Sanitaria Puerta de Hierro-Segovia de Arana (IDIPHISA), 28222 Majadahonda, Spain; 4 Research Unit, Primary Care Management, Madrid Health Service, 28035 Madrid, Spain; milagros.rico@salud.madrid.org; 5 Research Network on Chronicity, Primary Care and Health Promotion (RICAPPS-RICORS), Instituto de Salud Carlos III, 28029 Madrid, Spain; 6 Gregorio Marañón Health Research Institute, Madrid Health Service, 28007 Madrid, Spain; 7 Nursing Department, Faculty of Nursing, Physiotherapy and Podiatry, Complutense University of Madrid, 28040 Madrid, Spain; 8 UCM Research Group “Public Health-Lifestyles, Nursing Methodology and Care in the Community Environment”, Complutense University of Madrid, 28040 Madrid, Spain; 9 Department of Nursing, Physiotherapy and Medicine, University of Almería, 04120 Almería, Spain; ferplaza@ual.es; 10 Department of Nursing, Physiotherapy and Occupational Therapy, Faculty of Physiotherapy and Nursing, University of Castilla-La Mancha, Toledo Campus, 45071 Toledo, Spain; maria.ugarte@uclm.es

**Keywords:** migrants, social imaginaries, Primary Health Care, nurses, qualitative research, health equity, cultural humility, structural competence, racism, social determinants of health

## Abstract

**Highlights:**

**What are the main findings?**

- Social imaginaries about migrants among Primary Care nurses may operate as invisible relational barriers to access and care.
- Migration trajectories were understood in contrasting ways: as clinically irrelevant information or as central for contextualizing care.

**What are the implications of the main findings?**

- Improving accessibility requires interventions that address culturalist attributions and professional representations, not only formal administrative barriers.
- Training should bring professionals closer to migrant narratives and promote culturally sensitive, compassionate, anti-racist, and structurally informed practice.

**Abstract:**

**Background/Objectives:** Primary Health Care is central to universal health coverage and equity. Nurses can facilitate effective accessibility, but their social imaginaries may influence clinical interaction and the perceived legitimacy of migrants’ demands. The aim of this study was to understand how Primary Care nurses construct social imaginaries about migrants. **Methods:** An interpretive qualitative study informed by a critical anthropological perspective was conducted. Eight Primary Health Care nurses participated. Purposive sampling was followed by snowball sampling. Between January and July 2025, in-person semi-structured interviews were audio-recorded, transcribed verbatim, and analyzed using thematic analysis. **Results:** Three themes were identified: (1) the construction of otherness; (2) the migration process; and (3) the “luck” of having healthcare and not being able to demand it. Participants’ discourses combined vulnerability and racialized differentiation with narratives of perceived abuse, deservingness, expected gratitude, and possible informal forms of access regulation. **Conclusions:** Across the three themes, social imaginaries simultaneously constructed migrants as vulnerable and as users suspected of abuse, linking these representations to judgments of deservingness and the legitimacy of healthcare demands. Primary Health Care nurses may act as facilitators or informal barriers to effective accessibility, supporting culturally sensitive, compassionate, anti-racist, and migrant-sensitive healthcare.

## 1. Introduction

International migration is a structural social process in contemporary societies. From the perspective of receiving countries, an immigrant is commonly understood as a person who moves to a country other than their nationality or usual residence, thereby making the destination country their new country of usual residence [1]. Contemporary migration is linked to economic globalization, border regimes, labor market demands, and public narratives about belonging, usefulness, and threat [2,3]. In this context, “the migrant person” is not only a demographic category but also a social figure produced through collective meanings.

This study uses the notion of social imaginaries as socially constructed schemes through which groups perceive, explain, and intervene in what they consider reality [4]. Within professional contexts, these broader social imaginaries may acquire situated expressions shaped by professional discourses, practices, and institutional settings, giving rise to professional imaginaries.

In receiving societies, the construction of otherness is often organized around difference, vulnerability, suspicion, and deservingness. In healthcare settings, these imaginaries may intersect with clinical practices, bureaucratic requirements, and professional judgments about who is considered a legitimate user of care [5,6,7,8]. Recent qualitative research has similarly shown that “difference” may be constructed through perceived patient characteristics, organizational conditions, and wider structural contexts; when difference is primarily attributed to stereotyped characteristics of migrant patients, it may contribute to processes of othering and reduce the responsiveness of care [9].

Public discourse on migration tends to racialize socioeconomic problems by attributing inequalities, precariousness, or pressure on services to migrants, rather than situating them within broader social and political processes [3,10,11]. The migration process involves more than spatial movement: it entails rupture, social repositioning, and new ways of negotiating health, illness, and care [12]. Research with migrant patients has shown the importance of exploring explanatory models of illness, expectations regarding care, communication needs, and barriers to equitable access to healthcare systems [13].

Primary Health Care is intended to be inclusive, effective, and central to universal health coverage, as well as to ensure accessibility and equity in care [14]. International recommendations on the health of refugees and migrants emphasize the need for competent professionals, diversity-sensitive systems, and strategies to reduce inequities [15]. In the Spanish and regional context, guidance for health and social care professionals also highlights the need to provide care to migrants in their diversity [16].

The social determinants of health framework links health equity to socioeconomic and political contexts, cultural norms, material resources, and axes of social stratification [17,18], and is consistent with recent Spanish and regional strategies emphasizing equity, universal access, and Primary and Community Health Care [19,20]. Within this framework, it is relevant to analyze not only the barriers faced by migrants but also the professional imaginaries that may shape accessibility, the clinical relationship, and the perceived legitimacy of their demands. However, intercultural care cannot be reduced to lists of cultural traits or rigid explanations about patients’ culture. Anthropological critiques warn that professional stereotypes and rigid models of “cultural competence” may obscure both lived experience and the structural conditions that function as axes of social inequality in health [21].

Although recent literature has increasingly addressed access barriers, intercultural communication, and the health of migrants, less is known about how Primary Health Care professionals construct social imaginaries about migrants [22]. Qualitative evidence from healthcare settings suggests that professionals’ attitudes, together with considerations regarding eligibility and healthcare costs, may influence access and prioritization even where a formal right to care exists [23]. Analyzing these imaginaries allows the focus to shift from a position centered exclusively on the barriers faced by migrants toward the institutional and relational processes that may condition accessibility, trust, and quality of care.

The aim of this study was to understand how Primary Care nurses construct social imaginaries about migrants. Within this overarching aim, the study explored the elements that configure these imaginaries, the influence of personal and professional experiences, and the ways in which these constructions may relate to the perceived legitimacy of healthcare use and to the clinical relationship with migrants. The analysis focused on how professional discourses produce meanings, social categories, and judgments of deservingness that may influence healthcare practice. This focus is aligned with current debates on migrant-sensitive healthcare, cultural humility, structural determinants of health, and the role of healthcare professionals as agents who may either reproduce or challenge relational, cultural, and structural barriers to care.

## 2. Materials and Methods

### 2.1. Design and Methodological Approach

An interpretive qualitative design informed by a critical anthropological perspective was adopted. This perspective understands meanings as situated, contextual, and co-constructed between participants and the researcher [24].

Reporting of the study was guided by the Consolidated Criteria for Reporting Qualitative Research (COREQ) checklist, a transparency standard for qualitative interviews [25]. The completed 32-item COREQ checklist is provided as Supplementary Table S1.

### 2.2. Setting

The study was conducted in a management area of the Castilla-La Mancha Health Service, Spain. Primary Health Care constitutes the first level of care in the Spanish National Health System and is organized into health centers and local clinics, from which longitudinal, preventive, community, home-based, acute, and chronic care is provided.

This management area includes thirty basic health zones, thirty health centers, one hundred local clinics, and twenty-seven continuing care points, serving approximately 520,919 people in 116 municipalities [26,27].

To contextualize participant selection, municipalities within the management area were reviewed using publicly available data on total population and population born abroad. This review made it possible to identify areas with a greater migrant population and to guide the invitation to professionals with direct clinical experience caring for this group.

The category “diversified intermediate”, derived from the regional classification of rural and depopulated areas, was used only as a contextual descriptor for the care setting, alongside urban and peri-urban settings.

### 2.3. Participants and Sampling

Inclusion criteria were being a Primary Health Care nurse in the Castilla-La Mancha Health Service, holding either a temporary or permanent position, having at least one year of experience in Primary Health Care, and working in areas where regular clinical contact with migrant populations was expected. Professionals holding managerial or trade union positions were excluded. The definition of a Primary Health Care nurse was consistent with Spanish regulations on basic health structures [28].

Sampling was initially purposive, considering demographic and care-related characteristics such as sex, age, years of experience in Primary Health Care, urban, peri-urban, or diversified intermediate setting, specialization in Family and Community Nursing, and type of population cared for—adult or pediatric. Snowball sampling was subsequently used to access additional professional profiles.

The principal investigator established initial contact with professionals working in areas with a significant migrant population, since participants were expected to contribute direct experience of the study phenomenon. There was no direct employment, care, or hierarchical relationship between the researcher and the participants. Nine professionals were invited to participate; one declined for personal reasons. Participation was voluntary and informed. The final sample consisted of eight professionals. Data collection ended when the expected profiles had been covered, and the final interviews reinforced the central thematic patterns without contributing substantively to new analytical findings. This decision was based on information power and thematic stability, without assuming theoretical saturation in a strict sense [29,30], as shown in Table 1.

### 2.4. Data Collection Techniques

Semi-structured interviews were conducted using an interview guide designed to explore the experiences, meanings, and perceptions of nurses regarding migrants, as well as the possible influence of these imaginaries on professional practice. The guide was developed based on the study’s aim, the literature review, and the initial analytical dimensions. The main axes were construction of the imaginary of the migrant person; personal and professional experiences; service use, barriers, and legitimacy of demand; and typologies of imaginaries and forms of social differentiation. These domains were intended to explore, respectively, the categories and representations through which migrants were constructed; the personal and professional experiences contextualizing these meanings; the possible relationship between such constructions, healthcare use, barriers, and the legitimacy attributed to demands; and the forms of differentiation through which distinct migrant groups were socially positioned. The full guide is included as Supplementary Materials (Table S2). The interview guide was not formally pilot tested. Consistent with the reflexive and flexible nature of semi-structured interviewing, its core domains were maintained throughout data collection, while follow-up questions and probes were adapted iteratively to participants’ responses.

All interviews were conducted in person by the principal investigator between January and July 2025. Only the researcher and the participant were present in each interview. Interviews were audio-recorded and subsequently transcribed verbatim. Participants chose the interview location, indicating that it should ensure sufficient privacy and adequate recording quality. Four interviews were conducted in health centers, two in cafés, and two in libraries, always in quiet spaces that allowed confidentiality to be maintained. Interviews lasted between 35 and 54 min.

Before each interview began, the researcher presented the study, explained its purpose, clarified the voluntary nature of participation, the possibility of withdrawing at any time, and the confidential handling of data. It was confirmed that participants had understood the information provided, and informed consent was obtained. Each professional participated in a single interview; no additional follow-up interviews were conducted. During and after fieldwork, reflexive and field notes were prepared to record impressions, decisions, and incidents relevant to the analysis.

### 2.5. Data Analysis

Data were analyzed using thematic analysis, following the phases proposed by Braun and Clarke [31]. The process included familiarization through repeated reading of the transcripts, generation of initial codes, grouping of codes into potential themes, review and refinement of themes, definition and naming of themes, and selection of representative extracts. Iterative comparisons were conducted between interviews, codes, and themes to identify convergences, divergences, and cases that nuanced the interpretation [32]. The analysis addressed both explicit content and the assumptions, ideologies, and social relations present in the discourses.

The concept of social imaginaries informed the analysis as an interpretive lens rather than as a predefined coding framework. Initial coding remained attentive to participants’ language and recurrent patterns in the data, while also examining how migrants were named, differentiated, evaluated, and positioned in relation to expectations about appropriate behavior, healthcare use, and professional responses. During theme development, these codes were grouped and interpreted in relation to broader patterns of meaning and social classification that organized participants’ understandings of migrants and healthcare encounters.

The principal investigator performed the initial coding and developed a first scheme of codes, categories, and themes. The analysis was organized using code matrices, analytical memos, and a record of interpretive decisions; no specific qualitative analysis software was used. This scheme was reviewed and discussed by the research team during the manuscript’s analytical development; interpretive differences were debated until the final thematic organization was agreed upon.

Vulnerability and deservingness were not explicitly addressed in the interview guide or predefined as thematic categories. They were developed during the iterative analysis as higher-order interpretive concepts. Vulnerability was identified in explicit participant statements and in recurrent descriptions of language barriers, precariousness, isolation, and difference. Deservingness was developed analytically from recurrent references to demandingness, abuse, and perceived contribution to the system. References to gratitude, adaptability, and the legitimacy of healthcare use also informed this concept. Both concepts helped connect patterns identified across the data. They were progressively refined alongside codes and themes as the analysis advanced. This development reflected the reflexive, iterative, and methodologically flexible character of the analytical process.

Categories were not assumed to be objective properties of participants, but rather constructions produced in the interview dialogue and situated within an institutional and social context. Transcripts and preliminary findings were not returned to participants for formal validation; analytical traceability was strengthened through illustrative quotations, reflexivity, and research team review. Quotations included in the Results section were translated from Spanish into English using ChatGPT (OpenAI; GPT-5.5 Thinking). as an AI-assisted translation tool. The resulting translations were subsequently reviewed against the original Spanish quotations to check semantic fidelity. No professional translation or formal back-translation procedure was undertaken.

### 2.6. Rigor, Reflexivity, and Researcher Positionality

Credibility was strengthened through verbatim transcription of interviews, repeated reading of transcripts, maintenance of a record of codes and analytical decisions, selection of quotations that allow readers to assess the relationship between data and interpretation, and contextual description of the sample and setting. Transferability was addressed by characterizing the care context, participant profiles, and institutional conditions in Primary Health Care. Dependability and confirmability were supported by an explicit analytical route and systematic reflexivity regarding the researchers’ positionality [33].

The principal investigator was a Primary Health Care nurse with prior training and experience in qualitative research applied to health, migration, and care. Except for one participant, the remaining participants did not know that the researcher shared the Primary Health Care professional field, although they did know that the study explored professional imaginaries about migrants. This insider position may have facilitated access, trust, and understanding of professional language, but it may also have influenced topic selection, question formulation, and the freedom with which participants expressed potentially problematic discourses.

To mitigate this risk, the analysis incorporated reflexive notes to identify preconceptions, discomforts, and potential blind spots. The COREQ checklist was used as a guide for transparency and quality in reporting interviews, not as a criterion for defining the study’s methodological orientation [25].

### 2.7. Ethical Considerations

The present study was conducted within the framework of a broader research project previously approved by the Clinical Research Ethics Committee of Hospital Universitario de Talavera de la Reina (approval code CEIm 7/2019; approved on 12 March 2019). The research reported in this manuscript forms part of that previously approved project and was therefore conducted under the same ethical approval. The study was conducted in accordance with the Declaration of Helsinki. Participants received oral and written information about the study and signed informed consent before participating.

Participation was voluntary, and participants were informed that they could decline to answer any question or to withdraw from the study without consequences. Interviews were anonymized using participant codes, and transcripts and analytical materials were managed confidentially. The fragments included in the manuscript were selected to avoid data that could directly or indirectly identify participants, centers, or patients.

The research did not involve foreseeable physical or psychological risks for participants, as it consisted of interviews about professional experiences and perceptions.

## 3. Results

Three interrelated thematic axes were identified: the construction of otherness, the migration process, and the “luck” of having healthcare yet being unable to demand it. Before each theme is presented, Table 2 summarizes the categories and their analytical meanings.

The identified themes describe ways of constructing migrants and show how these imaginaries, in participants’ narratives, relate to clinical interaction, decision-making, and the everyday management of care.

### 3.1. The Construction of Otherness

#### 3.1.1. Difference, Comparison, and Vulnerability

Participating professionals constructed migrants through a recurrent tension between being different and being similar. In several participants’ narratives, migrants were compared with Roma people, a historically stigmatized minority in Spain. Although Roma people were not included in the interview guide, they emerged spontaneously as a reference for interpreting conflict, adaptation, and professional strategies. This comparison shows how, in some discourses, migrants were placed alongside other groups perceived as “different,” while also being differentiated from a group considered more familiar because of prolonged coexistence.

“*We have more conflict with Roma people, who are less respectful, than with immigrants. (E5)*”

Vulnerability was another relevant descriptor. It was frequently linked to language barriers, precarious housing, social isolation, and difficulties navigating the healthcare system. However, vulnerability could also become a homogenizing category that implied a risk of paternalism in professional action and a loss of agency. One participant explicitly connected vulnerability with communication difficulties:

“*They are usually people who are in a vulnerable situation. There are differences, but one fundamental one is the language barrier. Sometimes it is very difficult to know what is happening to them or for them to tell you. (E1)*”

This fragment shows how vulnerability was expressed in the professional account through the difficulty of understanding and being understood during the consultation. Across the interview data, these constructions were associated with differences in communication styles, in the information prioritized, and in the degree of participation attributed to the person receiving care.

#### 3.1.2. Maghrebi and Muslim People as Salient Otherness

Although participants referred to diverse groups of migrants, Maghrebi and Muslim people appeared prominently in several accounts. Latin American people were sometimes described as culturally or linguistically closer, whereas Maghrebi people were positioned as more distant. These representations did not appear with the same intensity in all interviews, but they constituted a relevant axis of differentiation.

Participants linked this perceived cultural distance with communication and trust difficulties, as well as with expectations of conflict in the care relationship. Difference was produced through references to religion, family organization, everyday practices, and gender relations.

“*I would distinguish between Arab people and almost the other large group of the immigrant population. (E8)*”

“*With Arabs, Africans, Muslims, we “clash,” because we have nothing in common; religion is very different, their customs, their way of seeing the family, their way of seeing reality. (E6)*”

The interview material also showed the use of derogatory terms to refer to Moroccan people. This language was identified by some participants as an everyday and problematic way of naming this group in a professional setting.

“*People of Moroccan origin are still called Moors, always in a derogatory tone. (E1)*”

#### 3.1.3. Gender Imaginaries: Hostile Men, Submissive Women, and Positive Exceptions

In several accounts, Maghrebi men were described as difficult for female professionals, especially when the clinical relationship involved accepting care instructions, negotiating treatment decisions, or addressing bodily care. This imaginary was linked to assumptions about patriarchal gender relations and women’s subordination.

“*Them, because we are women. And so, that thing about a woman being above them, they do not take it well at all. (E7)*”

At the same time, some men were constructed as positive exceptions when they approximated the figure of the “collaborative patient” expected by professionals: communicative, caring, responsible, concerned about their family, and adherent to recommendations or care plans.

“*I have had spectacular fathers in pediatrics. One had just arrived; he managed quite well, explained everything, and said: “You need to see my wife; she is in pain, to see if you can get her an appointment with the doctor.” He was charming. (E8)*”

Women were often attributed ideas of dependence on male relatives, lack of interest in learning Spanish, or submissiveness. The veil appeared only occasionally, but when it did, it was interpreted with curiosity, sacredness, or difficulty in understanding the female body within professionals’ cultural frameworks. Across the interview data, these prior expectations could shape communication, trust, and the autonomy attributed to migrants receiving care.

“*They come much less often, and when they come, they are very submissive and very polite and so on. And the men seem not to trust women very much, what we might tell them. (E8)*”

### 3.2. The Migration Process

#### 3.2.1. From the Clinical Case to the Life Process

Participants differed in whether they considered the migration trajectory clinically relevant and in how to address it. For some, equal treatment meant not asking about the migration process, the reasons for migration, or the social context. This position tended to focus care on the clinical case and on the biomedical information considered necessary for care, such as clinical history, vaccinations, or allergies.

“*I do not ask how they arrived, not at all, because that is not of interest to me. I treat people; what interests me is their medical history, what vaccinations they have, what history they have, what allergies they have. (E3)*”

For other participants, the migration process was essential to understanding the person and adapting care.

“*When something as important as leaving your country, your origin, where you belong, for somewhere totally different, with another culture and another language, happens, it can affect you. It is really determinant; it is extremely important to ask. (E4)*”

Between these two poles, some professionals asked only when the person offered information spontaneously or when the situation seemed unusual, which aroused professional curiosity. Trust and longitudinal relationships were described as conditions that facilitated the emergence of migration narratives.

Across the interview data, these divergent positions were associated with diverse ways of understanding the anamnesis: while some professionals limited relevant information to biomedical data, others incorporated the migration trajectory to contextualize care and adapt interventions.

#### 3.2.2. Proximity, Care Work, and Exemplary Migration

When some professionals had close knowledge of migrants’ trajectories, especially through relationships with formal caregivers, discourse tended to shift from suspicion to recognition of effort, family obligations, debt, separation, and aspirations. Caregivers from different countries appeared in accounts as workers who sustained transnational families and sought a better future for their children. These narratives complicated prior stereotypes of passivity or dependence.

“*A caregiver used part of her salary for her parents near Casablanca, in Morocco. She had the burden there and the economic and family burden here. (E1)*”

“*She was a teacher in Peru and came to care for an older man because she wanted to provide education for her children. (E7)*”

Some professionals, with relatives who had migrated or with close contact with migrant caregivers, described migration as an emotionally significant and clinically relevant experience. However, in several participants’ narratives, personal experience could also favor an image of the “exemplary migrant,” associated with more regularized, orderly, or economically self-sufficient trajectories, which served as an implicit reference for evaluating other migration experiences.

“*Whenever she tells me about her migration process, it really affects me, because she had a very hard time. I always try to ask because it moves me personally. (E4)*”

### 3.3. The “Luck” of Having Healthcare and Not Being Able to Demand It

#### 3.3.1. Open Doors and the Language Barrier

Participants often described the Spanish healthcare system as open, free, and easily accessible. In these accounts, migrants were imagined as users with few barriers beyond language. The metaphor of “open doors” referred to the system’s formal availability, but it could obscure the fact that having a right to care does not necessarily mean being able to access, understand, and participate in it on equal terms.

“*I do not see that they do it with bad intentions. Anything that happens to them, they come because they see that it is so easy. We have open doors, and you care for them well. (E5)*”

Language difficulties were also repeatedly identified as a barrier to healthcare. Participants described several strategies to address communication problems. These included attending consultations with relatives or other people who could act as informal interpreters. This pattern suggests that, when direct communication was difficult, the burden of overcoming the language barrier could fall on patients and their informal support networks.

“*If they did not understand you, they tried to make sure someone was there to translate, but with others it was impossible, I’m telling you. (E7)*”

Taken together, these accounts suggest that perceptions of an open and easily accessible healthcare system could be related to informal judgments about which demands were considered reasonable, which difficulties warranted a flexible response, and when it was considered necessary to “set limits.”

#### 3.3.2. Demandingness, Abuse, and Deservingness

The possibility of accessing healthcare on equal terms generated discomfort in several participants’ narratives. Some practices of demand, rights-claiming, or complaint, also present in the general population, were more readily interpreted in migrant patients as abuse or as insufficient contribution to the financing of the healthcare system.

“*How quickly they learn. People in general are demanding, and that demandingness or intransigence rubs off on them very quickly. (E8)*”

“*I do not understand how a person who has just arrived has the same healthcare rights as a person who has lived in Spain all their life. (E3)*”

This theme showed the ambivalence between understanding universal healthcare as a right and, in several participants’ narratives, representing it as a benefit to be received with gratitude. Complaints, home-visit requests, requests for materials before traveling, and missed appointments were, in these accounts, associated with doubts about the legitimacy of certain demands or with expectations of greater gratitude.

#### 3.3.3. Rolling out the Red Carpet or Controlling Access

Participants described two professional positions: “rolling out the red carpet,” that is, facilitating care for people who arrive with difficulties understanding how the healthcare system works, without an appointment, or with communication barriers; and “setting limits,” that is, enforcing schedules, rules, and behaviors considered acceptable among users.

“*Colleagues have told me: “they come late, they come without an appointment, and you roll out the red carpet for them.” But I cannot help it; that is how I am, and it is not because of him, it is because of anyone who comes a bit lost. (E8)*”

In several participants’ narratives, the idea of “setting limits” was linked to the need to organize healthcare demand; however, when combined with stereotypes about migrant users, it could operate as an informal form of access regulation.

“*Some do come, and you have to set a bit of a limit, because they cannot come whenever they want and then be seen immediately. (E7)*”

In this sense, professional imaginaries were not limited to an abstract level. Across the interview data, they contributed to defining which demands, complaints, or forms of system use were considered legitimate.

## 4. Discussion

The analysis suggests that the social imaginaries constructed by Primary Health Care nurses about migrants were not homogeneous but ambivalent and shaped by social discourses, institutional routines, and individual experiences. The central tension was articulated between vulnerability and demandingness: migrants were described as vulnerable when language barriers, precariousness, isolation, or migratory suffering were emphasized, but they could be represented as demanding or abusive when they exercised rights, expressed expectations, or claimed care. The specific contribution of this study is to show how these meanings, in professional discourses, relate to the clinical interpretation of the person, the consideration of their migration trajectory, and the moral valuation of the legitimacy of their demands. Viewed through the framework of social imaginaries, the three themes illustrate how broader patterns of meaning concerning otherness, migration trajectories, and legitimate healthcare use become situated in professional classifications and moral judgments in everyday care.

Taken together, the three themes articulate vulnerability and deservingness as cross-cutting interpretive dimensions rather than as discrete thematic categories. In the construction of otherness, vulnerability was associated with language barriers, precariousness, isolation, and culturalized difference. Judgments about adaptability and behaviors considered appropriate also implicitly introduced criteria of deservingness. In the migration-process theme, knowledge of personal biographies and trajectories could foster a more empathetic and compassionate understanding and promote recognition of situations of vulnerability. However, it could also give rise to the figure of the “exemplary migrant” as a normative reference against which other migration trajectories were evaluated. In the third theme, deservingness became explicit through judgments about demandingness, abuse, perceived contribution to the system, gratitude, and the legitimacy of healthcare use. Thus, vulnerability and deservingness intersect across the three themes as relational and moral frameworks through which migrants and the legitimacy of their healthcare demands are interpreted.

The construction of otherness appeared through comparisons with historically stigmatized groups and through prior expectations about conflict, adaptation, and behavior. This finding is consistent with research conducted in European Primary Health Care, where encounters between professionals and asylum seekers were traversed by othering practices, stereotypes, mistrust, and insufficient care resources [34]. The spontaneous comparison with Roma people suggests that professional categories were not produced in isolation, but in dialogue with historical hierarchies of belonging and exclusion [35].

Furthermore, Dullat et al. described how institutional discrimination, language barriers, and negative interactions with different agents of the healthcare system can generate delays, emotional suffering, distrust, and disengagement from services among Roma populations [36].

Vulnerability was a central category, especially associated with language and with difficulties navigating the healthcare system. A pattern was also described in the literature on nursing practices directed at migrant populations in Primary Health Care [37]. However, the results suggest that vulnerability may be formulated in a homogenizing way if it is presented as an intrinsic characteristic of migrants, rather than linked to social, administrative, labor, residential, or legal conditions that limit the effective exercise of rights. From this perspective, it is necessary to recognize situations of vulnerability without reducing the agency of the people receiving care [38].

Studies on nursing and refugee health show that power in the care relationship is fluctuating: nurses may facilitate, coordinate, or filter access, while people receiving care negotiate, accept, reject, or seek alternatives within the system [39]. In this sense, an external and homogeneous categorization of certain “others” as vulnerable may foster victimization and legitimize regulatory interventions by groups with greater power over their lives [40].

The results also allow analysis of how racialization and racism can appear in everyday nursing practices and discourses. A recent qualitative study identified false assumptions, negative stereotypes, neglect, lack of credibility, and unequal treatment as manifestations of racism; in contrast, culturally humble care was characterized by asking questions, active listening, showing respect, attending to nonverbal communication, and individualizing care [41]. In our study, this dimension was observed in the use of derogatory terms, collective attributions, and the construction of “positive exceptions,” defined by their proximity to professional expectations of hygiene, compliance, gratitude, or responsibility.

The prominent presence of discourses about Maghrebi and Muslim people suggests a pattern of differentiation shaped by origin, religion, and gender. Representations of hostile men and submissive women could condition the expectations with which the care encounter began and homogenize diverse experiences [42,43,44]. Studies with Muslim women have shown that discourses about oppression, passivity, or lack of agency may be transferred to healthcare services and generate experiences of invisibility or stereotyped treatment [45]. Similarly, a qualitative review on maternity care for Muslim women concluded that negative experiences were explained not only by lack of cultural knowledge, but also by unconscious biases, inflexible care models, stereotypes, and insufficiently personalized care [46]. These findings reinforce the need to avoid rigid explanations based on patients’ “culture,” as cultural bias may essentialize migrants and make relevant social, economic, and biographical factors invisible [47].

In the Spanish context, this racialized differentiation is condensed especially in the figure of the “Moor,” a classificatory and stigmatizing category applied to Muslim people of Maghrebi origin [43].

Differences in the clinical consideration of the migration process reflect two distinct conceptions of equality. For some participants, treating everyone equally meant limiting the anamnesis to biomedical history and not asking about the reasons for migration, the journey, or living conditions after arrival. For others, knowing these elements allowed them to understand losses, family networks, transnational obligations, working conditions, and expectations about care. This tension has also been described among Primary Health Care nurses, who initially reported a uniform practice for migrant and non-migrant populations while recognizing differentiated needs and differing capacities to follow recommendations [48]. Similarly, professionals caring for young refugees distinguished between an instrumental orientation, focused on classifying and treating symptoms, and a holistic orientation, aimed at understanding the life, family, social, and migration trajectory [49].

Recognizing the migration process does not mean all health problems derive from migration or that the consultation is a systematic inquiry into potentially traumatic experiences. Its relevance depends on whether the information helps understand the reason for consultation, changes in health, care expectations, support networks, or difficulties in accessing and participating in care. In Primary Health Care, physicians and nurses have noted that knowing refugees’ journeys can modify the interpretation of symptoms, clinical priorities, and the coordination of support, although these narratives usually emerge gradually when there is a safe, longitudinal relationship [50].

The literature on care coproduction adds that integrating the trajectory does not consist of adding a section on migration to the medical record, but of reconstructing, together with the person, the sequence of events and relating it to medical, legal, residential, labor, family, and communication-related aspects [51].

The possibility of incorporating this information does not depend solely on the individual professional’s disposition. Qualitative studies in Primary Health Care show that limited time, an orientation toward immediate demand, insufficient funding, a lack of interpreters, and service fragmentation favor reactive care and hinder the exploration of psychosocial needs [52]. In Spain, professionals and migrant women cared for in sexual and reproductive health centers also identified a lack of mediation, insufficient practical training, and communication difficulties as obstacles to personalized care [53]. Therefore, equitable care requires avoiding two forms of reductionism: applying formal equality, which renders relevant circumstances invisible, and overemphasizing difference to the point of automatically attributing problems to culture or to a homogeneous migration trajectory.

The theme of the “luck” of having healthcare and not being able to demand it shows how the right to health may be reformulated as a benefit granted by the receiving society. Deservingness operates as a moral judgment situated between formal entitlement and effective access: need, identity, attitude, rule compliance, and the reciprocity attributed to the user may influence the way services are applied in everyday practice [54]. Among health professionals, this logic has been expressed through prioritization of those considered contributors, responsible, or vulnerable, and through transformation of care for people with precarious status into a privilege financed by taxpayers rather than a right [55]. From a human rights perspective, the legitimacy of healthcare demands should not depend on judgments about economic contribution, length of residence, perceived cultural adequacy, or gratitude expressed toward the healthcare system [56].

Deservingness assessments affect not only entry into the system but also the standard, timeliness, and quality of care. Analysis of clinical cases has shown that assumptions about which patients deserve investment may lead to delays, reduced credibility, lower-quality interventions, or exclusion from usual benefits [57]. In Sweden, professionals caring for undocumented migrants described how costs and fiscal contribution influenced judgments about who should receive care [23]; in the United Kingdom, restrictive migration policies placed professionals in tension between the duty of care and implicit control functions [58]. These antecedents allow the positions of “rolling out the red carpet” or “setting limits” to be interpreted as practices that may facilitate orientation and continuity but may also operate as informal barriers when applied selectively, based on stereotypes about abuse, contribution, or adaptation.

The results call into question the equivalence among free care, formal right, and effective accessibility. A systematic review conducted in Spain identified language, administrative, informational, and discriminatory barriers, as well as difficulties derived from labor, residential, and legal status [59]. Data also show lower overall use of hospital consultations, procedures, and medicines among the population born outside Spain, while higher self-reported use of emergency care and admissions may be compatible with difficulties in access and continuity of care [60]. These data contradict generalizations about overuse and suggest that the metaphor of “open doors” may conceal obstacles that are not visible from inside services.

These findings extend previous research by showing how previously described barriers, stereotypes, and difficulties in migrant healthcare become interconnected within the professional imaginaries of participating Primary Health Care nurses in everyday practice. Three related processes were identified. Otherness was constructed through vulnerability and culturalized difference. Migration biographies and trajectories could humanize care, but they could also provide normative references for “exemplary” migration. Healthcare demands were also subject to moral judgments about demandingness, perceived contribution to the system, gratitude, and appropriate use of services. This suggests that effective accessibility is shaped not only by formal entitlement and organizational barriers, but also by relational and moral judgments produced in routine clinical encounters. In this process, professionals may act as informal gatekeepers by influencing which healthcare demands are considered legitimate and how access to care is mediated.

### 4.1. Implications for Practice and Healthcare Organization

The findings suggest that professional imaginaries may function as relational, informal, and invisible barriers to effective access for migrants. Therefore, interventions should not focus solely on modifying individual attitudes. Improving accessibility requires action on administrative, linguistic, and organizational barriers, as well as on professional representations that shape which demands are considered legitimate [56]. At the professional level, training is needed in cultural humility, structural competence, clinical communication, racism, and reflective practice [21,41,61]. In addition to conventional intercultural training, reflective case analysis, sustained contact with migrants, and work with narratives of their migration trajectories may be useful. These strategies may challenge stereotypes and foster culturally sensitive and compassionate practice; close contact has been associated with a better professional relationship with migrants [47].

Exploration of the migration trajectory should be guided by clinical relevance, consent, and the person’s willingness to narrate, avoiding both blindness to social determinants and routine searching for trauma or culturalist attribution of problems. At the organizational level, work should be undertaken through an intersectoral approach, while ensuring professional interpreting services, intercultural mediation when relevant, continuity of care, and sufficient time to understand complex needs. The barriers described in Primary Health Care show that these conditions cannot depend solely on each professional’s goodwill [50,52,53].

Primary Health Care models that incorporate nursing roles in coordination, education, guidance, and connection to other services demonstrate the profession’s potential to improve accessibility and continuity, provided they have specific training, regulatory recognition, and stable funding [62]. Relational care indicates that linguistic or cultural concordance may facilitate trust, but it does not replace listening, communicative clarity, sensitivity, and continuity, which any professional can provide [63]. At the institutional level, explicit anti-discrimination policies, equity evaluation systems, and mechanisms for migrant participation and coproduction are needed. Safeguards should also be established to ensure that judgments about gratitude, contribution, or compliance do not condition the legitimacy of the demand or the standard of care. Normative recognition of the right to healthcare must be accompanied by uniform procedures and measures to address the administrative, linguistic, organizational, and discriminatory barriers that persist [60].

### 4.2. Reflexivity and Influence of the Insider Position

The researcher’s insider position is a central element for interpreting the results. Sharing the profession and care setting may have favored interviews with a common language and access to tacit meanings of practice, but it may also have generated social desirability bias or, conversely, greater permissiveness in verbalizing comments that are naturalized within the professional group. This dual condition of proximity and interpretive risk was addressed through reflexivity and analytical recording, although it does not replace triangulation with other researchers or with migrant participants. Reflexive notes were revisited throughout the analytical process to identify preconceptions, discomforts, and potential blind spots, and to question initial interpretations in light of the interview data. Therefore, the results should be read as a situated interpretation of professional discourses, not as a total characterization of the nursing collective.

### 4.3. Limitations

The sample included eight nurses from a single management area. Although sampling sought variation in sex, age, professional experience, care setting, specialization, and type of population cared for, the small sample may have limited the diversity of perspectives represented. Less frequent, divergent, or contradictory positions may therefore have been underrepresented. Sample adequacy was assessed through information power [29] and thematic stability rather than representativeness. Previous empirical research has shown that relatively small samples may be sufficient to identify recurrent thematic patterns under specific study conditions [30,64]. However, the findings of the present study should be understood as contextually situated. Their transferability to other Primary Health Care settings requires consideration of local organizational, demographic, and professional conditions. In addition, the study used a single data collection technique—semi-structured interviews—and a single perspective on the phenomenon: that of nurses. The absence of triangulation of techniques, observation of clinical encounters, and contrast with migrants receiving care limits the possibility of analyzing how these discourses translate into observable interactions. Although the AI-assisted translations were reviewed against the original Spanish quotations, the absence of professional translation and formal back-translation may have limited the preservation of some idiomatic or culturally embedded nuances.

The researcher’s insider position may also have influenced data generation and interpretation, despite the reflexive measures adopted throughout the study. Future studies should incorporate the perspectives of migrants, combine interviews with observations and documentary analysis, and compare different organizational contexts to explore how professional imaginaries influence accessibility, trust, and the quality of care.

## 5. Conclusions

The discourses analyzed show that Primary Health Care nurses constructed migrants through ambivalent imaginaries of difference, vulnerability, demandingness, and deservingness. Across the three themes, vulnerability could support recognition of specific needs, but it could also homogenize migrant experiences and hinder individualized care. Attention to migration biographies could foster empathy, while also generating normative models of “exemplary” migration. Judgments about demandingness, perceived contribution to the system, gratitude, and appropriate use of services could influence the legitimacy attributed to healthcare demands.

Effective accessibility depends not only on formal entitlement and organizational conditions, but also on relational and moral judgments in everyday care. Primary Health Care should promote reflective, culturally sensitive, anti-racist, and structurally informed practice. Healthcare needs and the social determinants of health—not geographical origin, perceived contribution, or expected gratitude—should guide access to and quality of care. Future research should include migrants’ perspectives, observational approaches, and diverse organizational contexts. This research should examine how professional imaginaries shape clinical interactions and access to care, and how they may operate as invisible barriers within healthcare services.

## Figures and Tables

**Table 1:** Characteristics of the participants.

Participant	Sex	Age (Years)	Care Setting	Family and Community Nursing Specialist	Years in Primary Health Care/Type of Care
E1	Woman	50–59	Diversified intermediate	Yes	>20/Adults
E2	Woman	25–34	Peri-urban	Yes	6–10/Adults
E3	Man	45–54	Diversified intermediate	No	11–20/Pediatric
E4	Woman	25–34	Diversified intermediate	Yes	1–5/Adults
E5	Woman	35–44	Peri-urban	No	6–10/Adults
E6	Man	25–34	Urban	Yes	1–5/Adults
E7	Woman	50–59	Urban	Yes	11–20/Adults
E8	Woman	35–44	Peri-urban	Yes	11–20/Adults

**Table 2:** Themes and categories identified through thematic analysis.

Theme	Categories	Analytical Meaning
The construction of otherness	Comparison with Roma people; vulnerability; Maghrebi and Muslim people as salient otherness; gender imaginaries.	Otherness was organized through comparisons and differentiations by origin, culture, religion, and gendered assumptions, as well as through attributions of vulnerability and judgments about adaptability.
The migration process	From the clinical case to the life process; proximity and care work; family and professional biographies; exemplary migration.	The migration trajectory was alternatively ignored, treated as curiosity, or integrated as a determinant of health and care. Personal and professional experiences shaped empathy and normative judgments.
The “luck” of having healthcare yet being unable to demand it	Open doors and the language barrier; demandingness; perceived abuse; rolling out the red carpet or setting limits.	Universal and “free” healthcare was sometimes framed as generosity rather than as a right, fostering narratives of deservingness and possible informal forms of access regulation.

Quotations are identified using code E followed by the participant number (E1–E8).

## Data Availability

The data presented in this study are available on request from the corresponding author due to ethical and privacy restrictions. The interview transcripts are not publicly available because they may contain contextual information that could compromise participant anonymity. The article includes anonymized extracts that support the findings. Additional anonymized data may be provided by the corresponding author upon reasonable request and subject to applicable ethical approvals.

## Supplementary Materials

The following supporting information can be downloaded at: https://www.mdpi.com/article/10.3390/healthcare14172803/s1, Table S1: Consolidated Criteria for Reporting Qualitative Research (COREQ) Checklist. Table S2: Semi-structured interview guide.
