# Supplementary material for: Between Vulnerability and Deservingness: Social Imaginaries About Migrants Among Primary Health Care Nurses
Source: Healthcare (Basel). 2026 Sep 1;14(17):2803. doi: 10.3390/healthcare14172803 (PMC13566288; doi:10.3390/healthcare14172803)
Supplement: Supplementary file 1 [file healthcare-14-02803-s001.zip › healthcare-4463989-supplementary.pdf]

Supplementary Materials

Supplementary Table S1

Consolidated Criteria for Reporting Qualitative Research (COREQ) Checklist

Manuscript: Between Vulnerability and Deservingness: Social Imaginaries about Migrants among Primary Health Care Nurses

The checklist below reports the 32 COREQ items for interviews and focus groups and indicates where each item is addressed in the current revised manuscript. Where an item was not performed, this is stated explicitly rather than treated as unreported. Researcher credentials are reported as RN, MA (Applied Anthropology).

No.	COREQ item	COREQ guide question	Information reported for this study	Location in revised manuscript
**Domain 1. Research team and reflexivity**				
1	**Interviewer/facilitator**	Which author/s conducted the interview or focus group?	All interviews were conducted in person by the principal investigator and first author, Ángel Martín-García.	Section 2.4, p. 6, lines 202–203
2	**Credentials**	What were the researcher's credentials?	RN, MA (Applied Anthropology).	Reported in Supplementary Table S1; professional role and qualitative-research experience: Section 2.6, p. 7, lines 270–277
3	**Occupation**	What was their occupation at the time of the study?	Primary Health Care nurse.	Section 2.6, p. 7, lines 270–271
4	**Gender**	Was the researcher male or female?	Male.	Reported in Supplementary Table S1
5	**Experience and training**	What experience or training did the researcher have?	Prior training and experience in qualitative research applied to health, migration, and care.	Section 2.6, p. 7, lines 270–271
6	**Relationship established**	Was a relationship established prior to study commencement?	There was no direct employment, care, or hierarchical relationship between the researcher and participants. One participant knew that the researcher shared the Primary Health Care professional field.	Sections 2.3 and 2.6; p. 5, lines 174–175; p. 7, lines 270–277
7	**Participant knowledge of the interviewer**	What did the participants know about the researcher?	Participants knew that the study explored professional imaginaries about migrants. Except for one participant, they did not know that the researcher shared the Primary Health Care professional field.	Section 2.6, p. 7, lines 272–274

No.	COREQ item	COREQ guide question	Information reported for this study	Location in revised manuscript
8	**Interviewer characteristics**	What characteristics were reported about the interviewer/facilitator?	The manuscript discusses the researcher's insider position and its possible effects on access, trust, professional language, topic selection, question formulation, social desirability, interpretation, and participants' freedom to express potentially problematic discourses. Reflexive notes were used to identify preconceptions, discomforts, and potential blind spots.	Section 2.6, p. 7, lines 270–281; Section 4.2, p. 16, lines 686–697
**Domain 2. Study design**				
9	**Methodological orientation and theory**	What methodological orientation was stated to underpin the study?	Interpretive qualitative design informed by a critical anthropological perspective; meanings understood as situated, contextual, and co-constructed. Data were analyzed using thematic analysis following Braun and Clarke.	Sections 2.1 and 2.5; p. 4, lines 135–141; p. 6, lines 218–233
10	**Sampling**	How were participants selected?	Purposive sampling was initially used to seek variation in sex, age, Primary Health Care experience, care setting, Family and Community Nursing specialization, and adult/pediatric care profile; snowball sampling was subsequently used.	Section 2.3, p. 5, lines 167–171
11	**Method of approach**	How were participants approached?	The principal investigator established initial contact with professionals working in areas with a significant migrant population. Snowball sampling was subsequently used to access additional professional profiles.	Section 2.3, p. 5, lines 172–178
12	**Sample size**	How many participants were in the study?	Eight Primary Health Care nurses participated.	Section 2.3 and Table 1; p. 5, lines 177–183
13	**Non-participation**	How many people refused to participate or dropped out? Reasons?	Nine professionals were invited; one declined for personal reasons. No dropouts were reported after enrolment.	Section 2.3, p. 5, lines 176–178
14	**Setting of data collection**	Where was the data collected?	Four interviews were conducted in health centers, two in cafés, and two in libraries. Participants selected the location, provided that privacy and adequate recording quality could be ensured.	Section 2.4, p. 6, lines 202–208
15	**Presence of non-participants**	Was anyone else present besides participants and researchers?	No. Only the researcher and the participant were present during each interview.	Section 2.4, p. 6, lines 202–203
16	**Description of sample**	What are the important characteristics of the sample?	Sex, age, care setting, Family and Community Nursing specialization, years of Primary Health Care experience, and type of population cared for are reported for each participant.	Section 2.3, Table 1, p. 5

No.	COREQ item	COREQ guide question	Information reported for this study	Location in revised manuscript
17	**Interview guide**	Were questions, prompts, guides provided? Was it pilot tested?	Semi-structured interviews used a guide developed from the study aim, literature review, and initial analytical dimensions. The complete guide is Supplementary Table S1. The guide was not formally pilot tested. Its core domains were maintained, while follow-up questions and probes were adapted iteratively to participants' responses.	Section 2.4, pp. 5–6, lines 184–201; Supplementary Table S2
18	**Repeat interviews**	Were repeat interviews carried out?	No repeat or follow-up interviews were conducted.	Section 2.4, p. 6, lines 213–214
19	**Audio/visual recording**	Did the research use audio or visual recording?	All interviews were audio-recorded and subsequently transcribed verbatim.	Section 2.4, p. 6, lines 202–204
20	**Field notes**	Were field notes made during and/or after the interview or focus group?	Yes. Reflexive and field notes were prepared during and after fieldwork to record impressions, decisions, and incidents relevant to the analysis.	Section 2.4, p. 6, lines 214–216
21	**Duration**	What was the duration of the interviews or focus group?	Interviews lasted between 35 and 54 minutes.	Section 2.4, p. 6, line 208
22	**Data saturation**	Was data saturation discussed?	The study does not claim theoretical saturation in a strict sense. Data collection ended after the expected profiles had been covered and the final interviews reinforced central thematic patterns without substantively new analytical findings. Sample adequacy was assessed through information power and thematic stability.	Section 2.3, p. 5, lines 178–182; Section 4.3, pp. 16–17, lines 703–712
23	**Transcripts returned**	Were transcripts returned to participants for comment and/or correction?	No. Transcripts and preliminary findings were not returned to participants for formal validation.	Section 2.5, p. 7, lines 251–255
**Domain 3. Analysis and findings**				
24	**Number of data coders**	How many data coders coded the data?	The principal investigator performed the initial coding. The resulting code/category/theme scheme was reviewed and discussed by the research team, and interpretive differences were debated until agreement on the final thematic organization.	Section 2.5, pp. 6–7, lines 234–239
25	**Description of the coding tree**	Did authors provide a description of the coding tree?	The manuscript describes the development of a scheme of codes, categories, and themes, supported by code matrices, analytical memos, and a record of interpretive decisions. Table 2 summarizes themes, categories, and analytical meanings.	Section 2.5, pp. 6–7, lines 234–239; Results, Table 2

No.	COREQ item	COREQ guide question	Information reported for this study	Location in revised manuscript
26	**Derivation of themes**	Were themes identified in advance or derived from the data?	Themes were developed iteratively from the data. Vulnerability and deservingness were not predefined thematic categories; they were progressively developed as higher-order interpretive concepts alongside codes and themes.	Section 2.5, p. 7, lines 240–250
27	**Software**	What software, if applicable, was used to manage the data?	No specific qualitative analysis software was used.	Section 2.5, p. 6, lines 235–236
28	**Participant checking**	Did participants provide feedback on the findings?	No formal participant checking was undertaken. Analytical traceability was strengthened through illustrative quotations, reflexivity, and research-team review.	Section 2.5, p. 7, lines 251–255
29	**Quotations presented**	Were participant quotations presented to illustrate themes/findings? Was each quotation identified?	Yes. Participant quotations are presented throughout the Results and identified by code E1–E8. Quotations were translated from Spanish into English using an AI-assisted tool and reviewed against the original Spanish for semantic fidelity.	Results, p. 8, lines 306–308 and Sections 3.1–3.3; Section 2.5, p. 7, lines 256–260
30	**Data and findings consistent**	Was there consistency between the data presented and the findings?	Yes. The three themes and their analytical meanings are summarized in Table 2 and supported by illustrative participant quotations and interpretive text throughout the Results.	Results, Table 2 and Sections 3.1–3.3, pp. 8–12
31	**Clarity of major themes**	Were major themes clearly presented in the findings?	Yes. Three major themes are explicitly identified and organized as separate Results subsections.	Results, p. 8, lines 301–308; Sections 3.1–3.3
32	**Clarity of minor themes**	Is there a description of diverse cases or minor themes?	Yes. Subthemes and categories are reported under each major theme, and Table 2 provides an overview of categories and analytical meanings. Divergent positions are also described, for example regarding whether the migration trajectory is clinically relevant.	Results, Table 2 and Sections 3.1.1–3.3.3, pp. 8–12

Table S2. Semi-structured interview guide

Interview guide domains	Topics explored	Examples of guiding questions
Construction of social imaginaries about migrants	Images and meanings associated with migrants; economic migration; transnational families; discourses on the use or abuse of the healthcare system; the principle of difference; equal/equitable treatment; the migration process; cultural competence and cultural humility; training to address racism.	"I would like you to tell me about your day-to-day experience of caring for migrants." "How would you describe the migrants you usually care for?" "How do they usually present? What do they request, and what information do you usually collect?" "When caring for a migrant, do you ask about their migration trajectory or what they experienced before arriving here? In what circumstances?"
Personal and professional experiences	Personal, family, or other close experiences of migration; working alongside migrant professionals; community, neighborhood, association-based, or family contact with migrant populations; influence of these experiences on professional perspectives.	"I would like you to tell me whether you have had any personal, family, or close experience related to migration." "Have you worked alongside colleagues of migrant origin?" "Beyond clinical practice, do you have any other type of contact with migrant populations?" "Do you think these experiences influence the way you perceive or care for migrants? Could you give an example?"
Use of services, barriers, and legitimacy of healthcare demands	Perceived use of the healthcare system; perceived frequent attendance or abuse of services; language, administrative, social, or cultural barriers; social determinants of health; racism and discriminatory treatment; legitimacy attributed to healthcare demands.	"How do you manage when caring for migrants in your consultation?" "What difficulties do you encounter most often?" "How are language or communication barriers managed?" "What is care like during out-of-hours shifts?" "Have you witnessed or experienced any situation that you would interpret as discriminatory treatment?"
Types of social imaginaries and forms of social differentiation	Processes of social differentiation linked to origin, religion, gender, and culture; Islamophobia and Maurophobia (morofobia); stereotypes about specific migrant groups; attribution of individual behaviors to presumed collective cultural characteristics.	"Which cases have you found most difficult to manage in your experience of caring for migrants?" "What do you think made those situations difficult?" "Do you think origin, culture, religion, or gender influence the care relationship in any way? Could you give an example?" "In your experience, is there a tendency to differentiate between or classify migrants according to their origin or cultural characteristics?"
